# Resistant Protein: Forms and Functions

**DOI:** 10.3390/foods11182759

**Published:** 2022-09-08

**Authors:** Emanuele Zannini, Aylin W. Sahin, Elke K. Arendt

**Affiliations:** 1 School of Food and Nutritional Sciences, University College Cork, College Road, T12 K8AF Cork, Ireland; 2 APC Microbiome Institute, T12 K8AF Cork, Ireland

**Keywords:** resistant protein, protein digestibility, protein structure, food structure, food design

## Abstract

Several global health risks are related to our dietary lifestyle. As a consequence of the overconsumption of ultra-processed and highly digestible protein (150–200% of the recommended value), excess dietary proteins reach the colon, are hydrolysed to peptides and amino acids by bacterial proteases and fermented to various potentially toxic end products. A diet reformulation strategy with reduced protein content in food products appears to be the most effective approach. A potential approach to this challenge is to reduce food digestibility by introducing resistant protein into the diet that could positively influence human health and gut microbiome functionality. Resistant protein is a dietary constituent not hydrolysed by digestive enzymes or absorbed in the human small intestine. The chemical conformation and the amino acid composition strictly influence its structural stability and resistance to in vivo proteolysis and denaturation. Responding to the important gap in our knowledge regarding the digestibility performance of alternative proteins, we hypothesise that resistant proteins can beneficially alter food functionality via their role in improving metabolic properties and health benefits in human nutrition, similar to fibres and resistant starches. A multidisciplinary investigation of resistant protein will generate tremendous scientific impact for other interlinked societal, economic, technological and health and wellbeing aspects of human life.

## 1. Introduction

Food systems are under pressure to feed the world’s growing population within the planetary boundaries while ensuring the livelihoods of millions of people working along the food chain from farm to fork and the sector’s environmental sustainability [1]. With a projected population growth of 9.6 billion by 2050 and 10.6 billion by 2100, the global appetite for meat and animal products will increase by 76% by 2050 [1]. Addressing this will necessitate more sustainable production of protein sources for human and animal nutrition. Future food farming systems targeting microalgae, single-cell protein, insect larvae and cellular agriculture can secure the production of alternative food/protein sources in a closed environment with consistent and efficient production performance [2]. Contrarily, plant-sourced foods/proteins—mainly sourced from cereals and legumes and produced from conventional farming systems—are exposed to biotic (pathogens, pests), abiotic (climate variability and change and extreme weather event) and institutional (food trade restrictions) risk factors [3]. However, due to their intimate interaction with the environment, plant-sourced foods represent our best option for mitigating biotic and abiotic pressures and regenerating our natural resources. To achieve this, we need to move strategically from the green to gold agriculture revolution making novel, synthetic systems in crop plants (i.e., enhance the efficiency of photosynthesis systems by improving the carbon fixation reaction), which will boost agriculture production and secure food for the future generation. This improvement in natural resource usage efficiency [4,5,6] has the potential to deliver a step-change in agricultural output. On the other hand, environment-disconnected food systems have the potential to deliver risk-resilient diets but hardly directly address the climate challenges, biodiversity losses or support agriculture sectors and resilient landscapes.

Individuals in affluent societies consume more calories than they burn, partially caused by energy-rich food products, resulting in obesity and associated pathologies [7]. According to the World Health Organisation, non-communicable diseases (NCDs) are the leading cause of death (86%), disease (77%) and disability in Europe [8]. NCDs are largely preventable, and many initiatives are exploring prevention and control. The magnitude of western overconsumption of food surpasses that of food wasted in the household [9]. In this scenario, protein is significantly more critical than fats and carbohydrates, both numerically and environmentally, because the average highly digestible protein intake in many Western countries is 150–200% of the recommended value [10,11]. Protein overconsumption (i.e., protein that is nutritionally unnecessary) in western countries has been widely reported [11,12,13] and is far above the Population Reference Intake (PRI) [14]. There is a clear rationale to decrease the daily intake of protein since a substantial body of evidence associates the overconsumption of protein with adverse effects on human health, such as disorders of bone and calcium homeostasis, renal and liver dysfunction, increased cancer risk, hyperalbuminemia and precipitated progression of coronary artery disease [15,16,17,18,19,20]. Refs. [21,22], therefore, suggest a ‘reversed’ diet transition by ‘using less’ (e.g., leaving the meat out of the dish) or ‘doing things differently’ by a diet reformulation strategy, with reduced protein content in food products appears to be the most effective approach. However, plans to convince free and affluent societies to eat healthy but not innately desired food have been largely unsuccessful in the past [15,16,17,18,19,20,21,22]. Since the beginning of nutritional science, it has been hypothesised that the nutrients ingested through our diet are not entirely absorbed in the body, and only part of them are available. In such context, the terms “(bio)availability and (bio)accessibility” has come into use to identify such proportions [17]. The relatively recent recognition of incomplete protein digestion and absorption, mainly from vegetables, raises interest in non-digestible protein fractions [23]. These fractions may safely be called “resistant proteins” and are neither absorbed within the small intestine nor hydrolysable by mammalian digestive enzymes in the small intestine but may confer additional physiological benefits beyond the classical nutritive function of the protein. Despite the absence or presence of entanglements with nonprotein ingredients, some researchers [24,25,26,27,28] consider resistant proteins as proteinous dietary fibre and include them within the dietary fibre definition along with celluloses, hemicelluloses, lignins, oligosaccharides, pectins, gums and waxes, resistant starches and associated compounds such as polyphenols [29]. When incorporated into future foods, resistant proteins can impact other dietary components’ behaviours in food matrices, specifically carbo [30]. However, their technological potential and metabolic and physiological effects remain almost unstudied.

## 2. Resistant Plant Protein: Functions

Digestibility and amino acid composition have been recognised as essential factors for evaluating dietary protein quality [31]. For this reason, most legume proteins accumulated in seeds are still considered inferior in quality to animal protein, even though they have a physiological role far beyond the provision of essential amino acids with unexpected nutritional significance. In such regard, preliminary studies have shown how resistant proteins may exert physiological functions similar to dietary fibre as per se or through the interaction with other dietary constituents such as resistant starch by modulating its fermentation pattern in the large intestine with the increase of the short-chain fatty acid content [30] and the modulation of the gut microflora performance [30,32]. Besides containing small peptides and easily digestible proteins, legumes contain protein fractions that are either partially or entirely resistant to human digestive enzymes. These peptides and proteins may provide significant physiological and health-promoting effects, notably cholesterol-lowering activity [21,22], protecting cardiovascular health, reducing inflammation and cancer risk, weight control [33] and increased insulin sensitivity [34,35]. On the other hand, their structural stability has been reported to affect in vivo digestibility and availability of essential amino acids and the production of bioactive compounds. In addition, structural traits of legume proteins are of primary importance for their potential allergenicity and toxicity. These adverse effects must be carefully considered to exploit the beneficial effects of proteins and peptides from legume seeds [36]. Known classes of non-digestible bioactive legume protein and peptides are (i) storage proteins 7S and 11S, globulin, prolamin, glutenins from soybean and lupin with ACE-inhibitory properties, hypotensive, anticarcinogenic and anti-inflammatory activities [37,38,39]; (ii) lectins (carbohydrate-binding proteins) characterised by a tight β-sandwich structure that allows them to survive the acidic environment of the digestive tract where lectins exert anti-cytotoxic and anticancer activities [40]; (iii) glycated pea storage protein that is able, at least partially, to escape digestion and act as a modulator of the bacterial metabolic activities and their adhesive potentials [41]; (iv) α-amylase inhibitor from the white bean as an active agent in weight loss and glycaemic control [42]; and (v) protease inhibitor with anticarcinogenic activities [43]. Amylase and protease inhibitors are a heterogenous group of organic molecules, including proteins (>15 kDa) and peptides (<15 kDa), and are usually used by plants as defence strategies against pathogens, such as viruses, bacteria, or herbivores [44,45]

**Figure 1:**
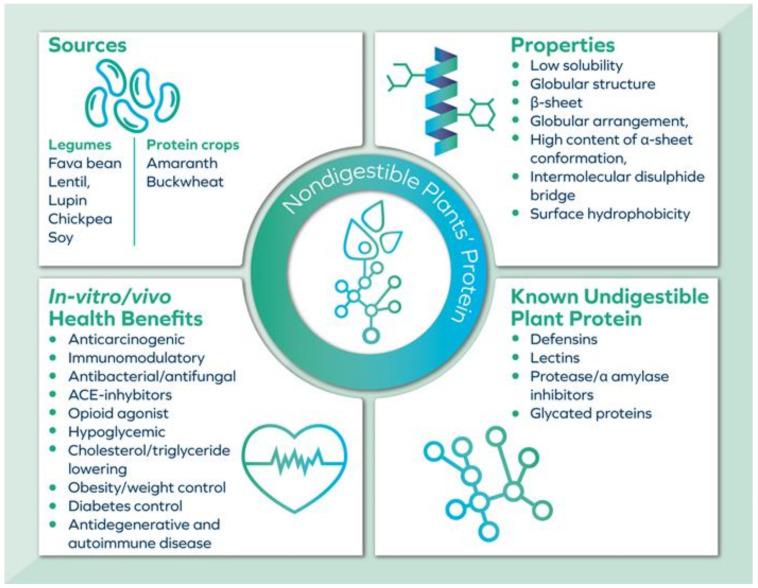
Principal physicochemical and biological properties of known classes of indigestible plant protein.

The compact structural feature of the protease inhibitor appears to have significant beneficial effects [46]. The main characteristics of the known undigestible proteins are depicted in Figure 1. Their structural peculiarity, interaction with other food constituents and low solubility are mainly responsible for their high stability and low digestibility [23].

Lectin, defensins, glycated protein and protein inhibitors have been widely investigated for their biological activities and represent the minor components of the non-digestible storage proteins/peptides. On the contrary, resistant protein, the principal constituent of the non-digestible storage protein, has been neglected. Therefore, its role in food design and human health has not yet been elucidated. Up to now, the full potential of resistant protein in food applications and human health enhancement remains untapped. Usually, plant-based resistant protein is separated during the industrial plant protein extraction process and discarded within the “fibre” side stream fraction. Over the past ten years, the scientific and industrial communities have focused on producing protein ingredients with high digestibility and optimal amino acid composition along with desired structure, rheology, palatability, flavour and appearance. However, much interest has recently been aroused in the new physiological function of these classes of proteins. From a physiological perspective, this new class of resistant protein can be proposed to be analogous to dietary fibre, potentially without the detrimental effects of some of the poorly absorbed fermentable oligo-, di-, monosaccharides and polyols (FODMAPs), highly present in the fibre fraction [47,48,49] and linked to irritable bowel syndrome (IBS) [50]. Moreover, the concept of digestible and indigestible proteins may be applied if a high concentration of amino acids in the plasma are detrimental to the patients—such as in metabolic genetic disorders (PKU), kidney deficiency or hepatic encephalopathy.

## 3. Resistant Plant Protein: Forms

The indigestibility trait of the resistant protein may originate from its structural peculiarities such as hydrophobicity, tertiary architecture characterised by a high content of β-sheet configuration, molecular conformation [51], the presence of thermally stable crosslinking formed by intra-and intermolecular hydrogen bonds and disulfide bridges [51] and its interaction with other food constituents such as carbohydrates [41,52]. Except for intrinsically occurring indigestible proteins, food processes applied during protein extraction/protein fractionation (acid or alkaline treatment) or food formulations (extrusion, boiling/cooking, fermentation) might build up indigestible protein species through aggregation, denaturation, polymerisation [51] and entanglement of proteins [23]. In investigating the effect of a highly resistant protein diet on young pig gut microbiomes, growth rate and metabolic profile, Murray and colleagues [53] manufactured a resistant protein diet by heating the feed (15 h at 70 °C followed by 20 min at 121 °C) to drive the Maillard chemistry of proteins and carbohydrates and confer digestive resistant status to the protein. The heat treatment of the resistant protein diet was designed to simulate the high heat processing that many ultra-processed food products undergo a concomitant development of resistant proteins. However, further investigation needs to be performed to provide robust evidence on such protein structure evolution. Similar to fibre components, resistant dietary proteins could have a disruptive effect on food structure by increasing matrix viscosity mainly due to their low water solubility, as previously reported for Marama bean proteins characterised by high β-sheet conformation hydrophobic interactions and tyrosine crosslinks [54]. Therefore, the inclusion of resistant protein in food formulation has to be adequately assessed regarding its potential structural interference with the food matrix architecture, rheology, colour, taste and appearance. However, much more investigation needs to be performed on these proteins’ physiological and nutritional significance in promoting the gut microbiota’s eubiosis condition that strongly influences our health and disease status.

## 4. Limitations

In this paper, the authors present and link preliminary data that need to be further validated with better animal models (e.g., growing pigs) or human clinical trials. Additionally, the fate of resistant protein passing into the colon requires an extensive investigation considering the positive and negative systemic and metabolic effects of colonic protein fermentation on the host [55]. In such regards, the resistant protein could reach the colonic microbiota and act as an amino acid source for protein fermenters, mainly species from *Clostridium*, *Desulfovibrio*, *Peptostreptococcus*, *Acidaminococcus*, *Veillonella*, *Propionibacterium*, *Bacillus*, *Bacteroides* and *Staphylococcus* [56,57]. In contrast to the extensively studied beneficial role of carbohydrate-derived short-chain fatty acids (SCFA), the effects of amino acid-derived SCFA on host physiology are not well known [58] and are associated with the production of other potentially harmful metabolites, including ammonia, sulfides and biogenic amines [59], among others with the potential capability to impact immunomodulatory, neurological, cardiovascular and gut functions [57,60,61]. These end-products may increase inflammatory response and tissue permeability and might be implicated in the development and severity of the symptoms of colorectal cancer and metabolic diseases, diabetes and non-alcoholic fatty liver disease [62]. A recent study conducted by Murray and colleagues [53] aimed to evaluate the effects of a standard vs. highly resistant protein diet on growth, gut microbiome, metabolomic profiles and the biomarkers of disease risk in pigs. The study demonstrates that the resistant protein was able to modulate the gut microbiome (metabolites) and negatively affect body mass and renal functions. Additionally, besides the potential health benefits of lectins, these sugar-binding proteins can bind to the surface of epithelial cells in the digestive system because of their high affinity for carbohydrates and can result in toxic reactions with changes in intestinal permeability [63,64]. In addition to the differences in protein digestibility due to protein source or processing factors, the variable capacities of individuals to lyse proteins (so-called digestive phenotype) may affect the abundance in which intact or partially degraded proteins are transferred to the large intestine [56].

## 5. Perspectives

**Figure 2:**
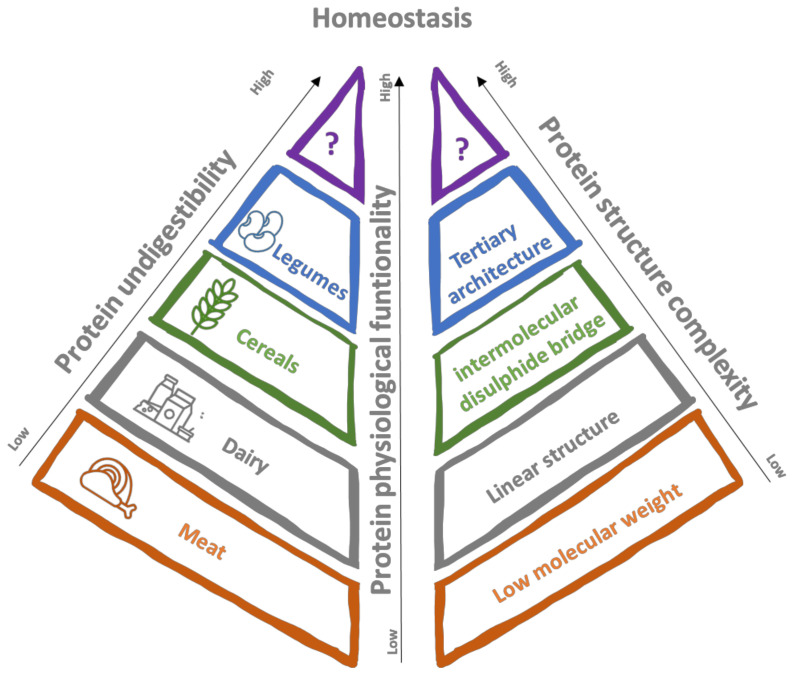
Overall representation of the proteinous fibre concept. The degree of protein indigestibility is strictly influenced by the raw materials where they are sourced and by the different food (bio)processing they undergo during their extraction, purification and subsequent inclusion in food products. However, learned scientist participation in this immature field is eagerly awaited.

The transition towards more inclusion of plant protein in our diets could bring us toward new and unexpected horizons regarding human health and wellbeing beyond the well-consolidated and known benefits. The investigation of the existence, distribution and physiological function of this class of proteinous dietary fibre could significantly contribute to longevity and public health, specifically in western countries where the increase in life expectancy is foreseen [40]. We suggest that the physiological significance of the resistant proteins, which are supposed to have only low nutritional value by the conventional nutritional assessment for protein, should be re-examined from this perspective (Figure 2).

Indeed, defining the new plant-resistant proteins and identifying their pre-and post-digestive multidisciplinary/interdisciplinary features will provide a crucial knowledge basis that will:

- Open a completely new research field on resistant protein, combining the interests of food scientists and food engineers to develop strategies for designing future foods.
- Advance the discipline of nutritional science by delivering comprehensive investigations elucidating the role of resistant protein in the maintenance of health and the prevention of non-communicable disease. The scientific advancement in this research field would significantly impact the future of food nutrition, public health and food policy development.
- Allow the food industry, in conjunction with national and international health authorities, to implement health promotion dietary strategies (personalised ingredients/foods) by establishing a pipeline in which the characterised resistant plant protein will be linked to NCD prevention for the potential as diet-based therapeutics.
- Endorse and facilitate a dietary shift to include resistant plant protein by consumers seeking a healthier, nutritious and more sustainable diet.

Every new finding brings new questions. For example, can we expect to identify these dietary proteins in other food sources such as macro/microalgae, insects and fungi? Are there striking differences among resistant proteins from different food sources? How do these protein categories modulate the physiological behaviour of the other food constituents in the large intestine? Can these proteins be ex situ synthesised? Can we use these proteins to design the future of food according to the citizen’s health requirements? Perhaps the first step is to elucidate further the physicochemical and biological peculiarities that characterised these new proteinous dietary fibre constituents.

## References

[1] United Nations (2012). United Nations Environment Programme.

[2] Parodi A., Leip A., De Boer I.J.M., Slegers P.M., Ziegler F., Temme E.H., Herrero M., Tuomisto H., Valin H., Van Middelaar C.E. (2018). The Potential of Future Foods for Sustainable and Healthy Diets. Nat. Sustain..

[3] Tzachor A., Richards C.E., Holt L. (2021). Future Foods for Risk-Resilient Diets. Nat. Food.

[4] Horton P., Long S.P., Smith P., Banwart S.A., Beerling D.J. (2021). Technologies to Deliver Food and Climate Security through Agriculture. Nat. Plants.

[5] Cavanagh A.P., South P.F., Bernacchi C.J., Ort D.R. (2022). Alternative Pathway to Photorespiration Protects Growth and Productivity at Elevated Temperatures in a Model Crop. Plant Biotechnol. J..

[6] Evans J.R., Lawson T. (2020). From Green to Gold: Agricultural Revolution for Food Security. J. Exp. Bot..

[7] Romieu I., Dossus L., Barquera S., Blottière H.M., Franks P.W., Gunter M., Hwalla N., Hursting S.D., Leitzmann M., Margetts B. (2017). Energy Balance and Obesity: What Are the Main Drivers?. Cancer Causes Control.

[8] World Health Organization (2012). Action Plan for Implementation of the European Strategy for the Prevention and Control of Noncommunicable Diseases 2012−2016.

[9] Alexander P., Brown C., Arneth A., Finnigan J., Moran D., Rounsevell M.D.A. (2017). Losses, Inefficiencies and Waste in the Global Food System. Agric. Syst..

[10] Aiking H. (2011). Future Protein Supply. Trends Food Sci. Technol..

[11] de Boer J., Aiking H. (2018). Prospects for Pro-Environmental Protein Consumption in Europe: Cultural, Culinary, Economic and Psychological Factors. Appetite.

[12] Hayashi K., Oita A., Lassaletta L., Shindo J., Shibata H., Sakurai G., Eguchi S. (2018). Reducing Nitrogen Footprints of Consumer-Level Food Loss and Protein Overconsumption in Japan, Considering Gender and Age Differences. Environ. Res. Lett..

[13] de Boer J., Aiking H. (2019). Strategies towards Healthy and Sustainable Protein Consumption: A Transition Framework at the Levels of Diets, Dishes, and Dish Ingredients. Food Qual. Prefer..

[14] EFSA NDA Panel (EFSA Panel on Dietetic Products, Nutrition and Allergies) (2012). Scientific Opinion on Dietary Reference Values for Protein. EFSA J..

[15] Mutlu E.A., Keshavarzian A., Mutlu G.M. (2006). Hyperalbuminemia and Elevated Transaminases Associated with High-Protein Diet. Scand. J. Gastroenterol..

[16] Delimaris I. (2013). Adverse Effects Associated with Protein Intake above the Recommended Dietary Allowance for Adults. ISRN Nutr..

[17] Southgate D.A.T., Johnson I.T., Fenwick G.R. (1989). Nutrient Availability: Chemical and Biological Aspects.

[18] Fleming R.M. (2000). The Effect of High-Protein Diets on Coronary Blood Flow. Angiology.

[19] Gautam B.P.S., Gondwal M., Kishore N., Kumar D., Kundapur R. (2015). Adverse Effect in Human Beings Associated with Excess Dietary Protein Intake. Biomedical Applications of Natural Proteins.

[20] Ko G.J., Rhee C.M., Kalantar-Zadeh K., Joshi S. (2020). The Effects of High-Protein Diets on Kidney Health and Longevity. J. Am. Soc. Nephrol..

[21] Sugano M., Goto S., Yamada Y., Yoshida K., Hashimoto Y., Matsuo T., Kimoto M. (1990). Cholesterol-Lowering Activity of Various Undigested Fractions of Soybean Protein in Rats. J. Nutr..

[22] Ogawa T., Gatchalian-Yee M., Sugano M., Kimoto M., Matsuo T., Hashimoto Y. (1992). Hypocholesterolemic Effect of Undigested Fraction of Soybean Protein in Rats Fed No Cholesterol. Biosci. Biotechnol. Biochem..

[23] Carbonaro M., Grant G., Cappelloni M., Pusztai A. (2000). Perspectives into Factors Limiting in Vivo Digestion of Legume Proteins: Antinutritional Compounds or Storage Proteins?. J. Agric. Food. Chem..

[24] Jones J.M. (2014). CODEX-Aligned Dietary Fiber Definitions Help to Bridge the ‘Fiber Gap’. Nutr. J..

[25] Davidson M.H., McDonald A. (1998). Fiber: Forms and Functions. Proceedings of the Nutrition Research.

[26] Calixto F.D.S. (1997). La Fibra Dietética En Nutrición y Salud. ANS. Aliment. Nutr. Salud.

[27] Trowell H. (1988). Dietary Fiber Definitions. Am. J. Clin. Nutr..

[28] Saura-Calixto F., Goñi I., Mañas E., Abia R. (1991). Klason Lignin, Condensed Tannins and Resistant Protein as Dietary Fibre Constituents: Determination in Grape Pomaces. Food Chem..

[29] Jiménez-Escrig A., Sánchez-Muniz F.J. (2000). Dietary Fibre from Edible Seaweeds: Chemical Structure, Physicochemical Properties and Effects on Cholesterol Metabolism. Nutr. Res..

[30] Morita T., Kasaoka S., Oh-hashi A., Ikai M., Numasaki Y., Kiriyama S. (1998). Resistant Proteins Alter Cecal Short-Chain Fatty Acid Profiles in Rats Fed High Amylose Cornstarch. J. Nutr..

[31] FAO (2013). Dietary Protein Quality Evaluation in Human Nutrition.

[32] Morita T., Kasaoka S., Kiriyama S. (2004). Physiological Functions of Resistant Proteins: Proteins and Peptides Regulating Large Bowel Fermentation of Indigestible Polysaccharide. J. AOAC Int..

[33] Kayashita J., Shimaoka I., Nakajoh M., Kato N. (1996). Feeding of Buckwheat Protein Extract Reduces Hepatic Triglyceride Concentration, Adipose Tissue Weight, and Hepatic Lipogenesis in Rats. J. Nutr. Biochem..

[34] He T., Giuseppin M.L.F. (2014). Slow and Fast Dietary Proteins Differentially Modulate Postprandial Metabolism. Int. J. Food. Sci. Nutr..

[35] Carbonaro M., Maselli P., Nucara A. (2015). Structural Aspects of Legume Proteins and Nutraceutical Properties. Food Res. Int..

[36] Verma A.K., Kumar S., Das M., Dwivedi P.D. (2013). A Comprehensive Review of Legume Allergy. Clin. Rev. Allergy Immunol..

[37] Jenkins D.J.A., Mirrahimi A., Srichaikul K., Berryman C.E., Wang L., Carleton A., Abdulnour S., Sievenpiper J.L., Kendall C.W.C., Kris-Etherton P.M. (2010). Soy Protein Reduces Serum Cholesterol by Both Intrinsic and Food Displacement Mechanisms. J. Nutr..

[38] Vital D.A.L., de Mejía E.G., Dia V.P., Loarca-Piña G. (2014). Peptides in Common Bean Fractions Inhibit Human Colorectal Cancer Cells. Food Chem..

[39] Scarafoni A., Magni C., Duranti M. (2007). Molecular Nutraceutics as a Mean to Investigate the Positive Effects of Legume Seed Proteins on Human Health. Trends Food Sci. Technol..

[40] Pryme I.F., Bardocz S., Pusztai A., Ewen S.W.B. (2006). Suppression of Growth of Tumour Cell Lines in Vitro and Tumours in Vivo by Mistletoe Lectins. Histol. Histopathol..

[41] Światecka D., Małgorzata I., Aleksander Ś., Henryk K., Elzbieta K. (2010). The Impact of Glycated Pea Proteins on Bacterial Adhesion. Food Res. Int..

[42] Barrett M.L., Udani J.K. (2011). A Proprietary Alpha-Amylase Inhibitor from White Bean (Phaseolus Vulgaris): A Review of Clinical Studies on Weight Loss and Glycemic Control. Nutr. J..

[43] Birk Y., Troll W., Kennedy A.R. (1993). Protease Inhibitors of Plant Origin and Role of Protease Inhibitors in Human Nutrition Overview. Protease Inhibitors as Cancer Chemopreventive Agents.

[44] Hellinger R., Gruber C.W. (2019). Peptide-Based Protease Inhibitors from Plants. Drug Discov. Today.

[45] Barbole R.S., Saikhedkar N., Giri A., Maheshwari V.L., Patil R.H. (2022). Plant Peptides as Protease Inhibitors for Therapeutic and Agricultural Applications. Natural Products as Enzyme Inhibitors: An Industrial Perspective.

[46] Wan X.S., Serota D.G., Ware J.H., Crowell J.A., Kennedy A.R. (2002). Detection of Bowman-Birk Inhibitor and Anti-Bowman-Birk Inhibitor Antibodies in Sera of Humans and Animals Treated With Bowman-Birk Inhibitor Concentrate. Nutr. Cancer.

[47] Atzler J.J., Ispiryan L., Gallagher E., Sahin A.W., Zannini E., Arendt E.K. (2020). Enzymatic Degradation of FODMAPS via Application of β-Fructofuranosidases and α-Galactosidases—A Fundamental Study. J. Cereal Sci..

[48] Ispiryan L., Zannini E., Arendt E.K. (2020). Characterization of the FODMAP-Profile in Cereal-Product Ingredients. J. Cereal Sci..

[49] Ispiryan L., Heitmann M., Hoehnel A., Zannini E., Arendt E.K. (2019). Optimization and Validation of an HPAEC-PAD Method for the Quantification of FODMAPs in Cereals and Cereal-Based Products. J. Agric. Food Chem..

[50] Halmos E.P., Gibson P.R. (2019). Controversies and Reality of the FODMAP Diet for Patients with Irritable Bowel Syndrome. J. Gastroenterol. Hepatol..

[51] Morrison J.J., Dayre Mcnally J., Navidzadeh A., Beauregard M. (2000). Development of an Optimized Feeding Technology for Dairy Cows. Appl. Biochem. Biotechnol..

[52] Finot P.-A. (2005). The Absorption and Metabolism of Modified Amino Acids in Processed Foods. J. AOAC Int..

[53] Murray M., Coughlan M.T., Gibbon A., Kumar V., Marques F.Z., Selby-Pham S., Snelson M., Tsyganov K., Williamson G., Woodruff T.M. (2022). Reduced Growth, Altered Gut Microbiome and Metabolite Profile, and Increased Chronic Kidney Disease Risk in Young Pigs Consuming a Diet Containing Highly Resistant Protein. Front. Nutr..

[54] Amonsou E.O., Taylor J.R., Emmambux M.N., Duodu K.G., Minnaar A. (2012). Highly Viscous Dough-Forming Properties of Marama Protein. Food Chem..

[55] Kårlund A., Gómez-Gallego C., Turpeinen A.M., Palo-Oja O.M., El-Nezami H., Kolehmainen M. (2019). Protein Supplements and Their Relation with Nutrition, Microbiota Composition and Health: Is More Protein Always Better for Sportspeople?. Nutrients.

[56] Dallas D.C., Sanctuary M.R., Qu Y., Khajavi S.H., van Zandt A.E., Dyandra M., Frese S.A., Barile D., German J.B. (2017). Personalizing Protein Nourishment. Crit. Rev. Food Sci. Nutr..

[57] Fuller M. (2012). Determination of Protein and Amino Acid Digestibility in Foods Including Implications of Gut Microbial Amino Acid Synthesis. Br. J. Nutr..

[58] Davila A.M., Blachier F., Gotteland M., Andriamihaja M., Benetti P.H., Sanz Y., Tomé D. (2013). Intestinal Luminal Nitrogen Metabolism: Role of the Gut Microbiota and Consequences for the Host. Pharmacol. Res..

[59] Gilbert M.S., Ijssennagger N., Kies A.K., van Mil S.W.C. (2018). Protein Fermentation in the Gut; Implications for Intestinal Dysfunction in Humans, Pigs, and Poultry. Am. J. Physiol. Gastrointest. Liver Physiol..

[60] Portune K.J., Beaumont M., Davila A.M., Tomé D., Blachier F., Sanz Y. (2016). Gut Microbiota Role in Dietary Protein Metabolism and Health-Related Outcomes: The Two Sides of the Coin. Trends Food Sci. Technol..

[61] Windey K., de Preter V., Verbeke K. (2012). Relevance of Protein Fermentation to Gut Health. Mol. Nutr. Food Res..

[62] Diether N.E., Willing B.P. (2019). Microbial Fermentation of Dietary Protein: An Important Factor in Diet–Microbe–Host Interaction. Microorganisms.

[63] He S., Simpson B.K., Sun H., Ngadi M.O., Ma Y., Huang T. (2018). Phaseolus Vulgaris Lectins: A Systematic Review of Characteristics and Health Implications. Crit. Rev. Food Sci. Nutr..

[64] Sudheendra C.V.K., Hazra T., Solanki A., Ramani V. (2021). Anti-Nutritive and Therapeutic Properties of Lectins. Biological and Chemical Hazards in Food and Food Products.

